# The clinical burden of newly diagnosed Heart failure among patients with Reduced, mildly Reduced, and preserved ejection fraction

**DOI:** 10.1016/j.ijcha.2023.101182

**Published:** 2023-02-14

**Authors:** Gregory A. Nichols, Qing Qiao, Anouk Déruaz-Luyet, Bettina J. Kraus

**Affiliations:** a Kaiser Permanente Center for Health Research, Portland, OR, United States; b Boehringer Ingelheim International GmbH, Ingelheim am Rhein, Germany; c Department of Internal Medicine I, University Hospital Würzburg, Germany

**Keywords:** Heart failure, Ejection fraction, Morbidity, Mortality

## Abstract

**Background:**

Contemporary analyses of the distribution of heart failure (HF) patients by groups of ejection fraction are not available or are limited to hospitalized patients. Our objective was to quantify the per-person and system level clinical burden of a broad population of HF patients.

**Methods:**

We studied 16,516 patients with a new HF diagnosis recorded in the electronic medical record of a U.S. integrated delivery system between 2005 and 2017. We used the diagnosis date as the index date and the nearest echocardiogram result to classify patients as HFrEF (n = 2,430), HFmrEF (n = 1,646), HFpEF (n = 12,440) and followed them through 2019 for major clinical outcomes (all-cause mortality, HF hospitalizations [HHF], all-cause hospitalizations, incident chronic kidney disease [CKD], progression of eGFR category, progression of CKD, incident type 2 diabetes [T2D], and progression to insulin use). We compared age and sex adjusted incidence rates and rate ratios of the outcomes between the HF types.

**Results:**

Incidence rates for most outcomes were significantly higher among patients with HFrEF compared with HFpEF. HHF was 59 % greater, mortality 31 % greater, and CKD incidence 55 % greater, (p < 0.001 for all comparisons). However, the larger size of the HFpEF group generated 4.7–6.7 times as many total outcomes.

**Conclusions:**

Regardless of subtype, the presence of HF was associated with poor clinical outcomes. Incidence rates were higher for HFrEF than HFpEF, but as the latter represented 75% of the study population, HFpEF caused a greater overall burden on the health care system, reflecting the high unmet need of target therapies for HFpEF.

## 1 Introduction

Heart failure (HF) is a major public health problem with a lifetime risk ranging from 20 % to 45 %.[1] The burden of HF is exacerbated by high rates of re-hospitalizations that exceed 20 % within 30 days of discharge and 60 % within 1 year.[2] In addition, HF is associated with other chronic diseases including cardiovascular disease (CVD), type 2 diabetes (T2D), and chronic kidney disease (CKD).[3] Heart failure is categorized by left ventricular ejection fraction (EF), and various definitions have been used. Recently, major international heart failure societies defined 4 groups in their universal classification for HF: HF with reduced EF (HFrEF, ≤40 %), mildly reduced EF (HFmrEF, 41–49 %), preserved EF (HFpEF, ≥ 50 %), and HF with improved EF (HFimpEF), the latter being newly introduced and defined as symptomatic HF with a baseline EF ≤ 40 %, a ≥ 10 point increase from baseline EF, and a second measurement of EF greater than 40 %.[4] The EF cut-points are now aligned with the American college of Cardiology (ACC)/American Heart Association (AHA)/Heart Failure Society of America (HFSA) categories.[5] Previous studies based on non-hospitalized patients have found an approximate equal prevalence of HFrEF and HFpEF, but all such studies were conducted before 2010.[6] This is critically important because studies reporting trends in HF consistently found a decrease in HFrEF coupled with an increase in HFpEF.[7], [8], [9] Thus, it is likely that HFpEF has come to represent a greater proportion of overall HF cases. We undertook the present study to evaluate the long-term risk of hospitalizations and mortality in a real-world population of patients in an integrated delivery system with newly diagnosed in inpatient, outpatient or emergency room encounters. In addition, because CKD and T2D are strongly associated with HF,[3] we examine the incidence and progression of CKD, and the incidence and progression of T2D.

## 2 Methods

### 2.1 Study site and data source

The study was reviewed by the Kaiser Permanente Northwest (KPNW) Institutional Review Board. Due to the observational study design, approval was granted with a waiver of informed consent. We conducted a longitudinal rolling cohort study using the electronic medical records (EMR) of Kaiser Permanente Northwest (KPNW), an integrated delivery system serving approximately 650,000 individuals in the 100-mile radius around Portland, Oregon. KPNW provides all medical services to its enrolled membership via hospitals, ambulatory clinics, pharmacies, and ancillary service owned and operated by the organization. These services are captured in an EPIC®-based EMR since 1996 that includes inpatient, outpatient, emergency, and radiology encounters. All services are linked seamlessly within the EMR, including laboratory results and a dispensing database that contains nearly all prescription medications received by members.

### 2.2 Sample selection and variables

For this study, we identified all 37,773 patients with an International Classification of Diseases, 9th Revision (ICD9) diagnosis of HF (428.x) recorded in the EMR between January 1, 2005 and September 30, 2015, or an ICD10 diagnosis (I51.x) recorded between October 1, 2015 and December 31, 2017. These diagnostic codes were extracted from all settings (inpatient, outpatient, and emergency), but were only included if they appeared as the primary reason for the encounter (first position). To ensure we were studying newly diagnosed HF, we required 12 months of health plan eligibility prior to the diagnosis and excluded 13,433 with prior mention of HF. For the remainder, we used the first HF diagnosis as the index date. From all available echocardiogram data, we selected the result nearest to and within 6 months before or after the index date to classify the remaining 24,340 patients as HFrEF (n = 2,430, 10.0 %), HFmrEF (n = 1,646, 6.8 %), HFpEF (n = 12,440, 51.1 %), or no echocardiogram available (n = 7,824, 32.1 %). We excluded those without an echocardiogram from the current analyses, most of whom did not have additional indications of HF, so the final analysis sample was 16,516 unique individuals (Supplemental Fig. 1). Characteristics of the included and excluded patients are compared in Supplemental Table 1.

Patients were followed from their first HF diagnosis (index date) until they died or left the health plan for other reasons, or until December 31, 2019. The baseline period for covariate data collection was during the 12 months preceding the index date. All covariate data, including demographics (age, sex, race/ethnicity), risk factors (blood pressure, body mass index [BMI], smoking status), cardiovascular disease (ICD-9: 410, 411, 412, 413, 414, 433, 434, 435 / ICD-10: I20, I21, I22, I25, G45, I63, I65, I66), diabetes (ICD-9: 250 / ICD10: E10, E11, E13), hypertension (ICD9: 401–405 / ICD10: I10-I16), pharmaceutical usage, and laboratory values were extracted from the EMR and linked datasets during the baseline data collection period.

### 2.3 Outcomes and statistical analyses

We independently examined eight clinical outcomes. Patients were included in all appropriate analyses, but only once per analysis. The first three outcomes were analyzed among all patients: 1) all-cause mortality, 2) hospitalization for heart failure, defined as an admission with a primary discharge diagnosis of HF; 3) hospitalization for any cause. In addition, we examined five clinical outcomes within selected subsets. These were 4) incident CKD, defined as two eGFR values < 60 ml/min/1.73 m^2^ during follow-up estimated from serum creatinine values per the 2009 CKD-*EPI* equation,[10] limited to those with a baseline eGFR ≥ 60; 5) worsening of eGFR category, defined as deterioration to a worse eGFR category among all patients; 6) worsening of CKD, defined as deterioration to a worse eGFR category during follow-up (category 3a, 45–59 ml/min/1.73 m2; 3b, 30–44; 4, 15–29; 5, <15 or ESKD), limited to those with CKD at baseline (2 eGFRs < 60); 7) incident T2D diagnosed during follow-up, limited to those without T2D at baseline; and 8) progression to insulin, defined as first use of insulin during follow-up, limited to those with baseline T2D without a prior insulin dispense.

We compared age and sex adjusted incidence rates and rate ratios of the outcomes between each of the HF types using generalized linear models with Poisson errors (log-link) and follow-up time as an offset variable (to account for differential follow-up). EF category was used as a class variable so that all three categories could be compared simultaneously in the same model. We explored models with additional covariates including BMI and eGFR but found consistent results. As an example, we display the results of models adjusting for age, sex, non-Hispanic Black race, smoking, history of atherosclerotic cardiovascular disease, blood pressure ≥ 140/90, and use of HF medications in supplemental table 2.

## 3 Results

Of the 16,516 patients in the primary analyses, 14.7 % (n = 2,430) had HFrEF, 10.0 % (n = 1,646) had HFmrEF, and 75.3 % (n = 12,440) had HFpEF (Table 1). Compared with HFrEF, those with HFpEF were older (72.2 vs 68.7 years) and more likely to be women (54.2 % vs 34.9 %). They were also more likely to have CKD (42.8 % vs 35.0 %), but less likely to have any cardiovascular disease (84.1 % vs 93.1 %). Systolic blood pressure was higher among patients with HFpEF (130 vs 121 mmHG) as was BMI (31.6 vs 29.1 kg/m^2^). In general, characteristics for patients with HFmrEF ranged between HFrEF and HFpEF.

**Table 1:** Demographic and clinical characteristics of 16,516 patients with diagnosed heart failure and an available echocardiogram by heart failure type.

	HF Type		
	HFrEF:	HFmrEF:	HFpEF:
	EF ≤ 40 %	EF 41–49 %	EF ≥ 50 %
Baseline Assessment	(n = 2,430)	(n = 1,646)	(n = 12,440)
% of Total	14.7 %	10.0 %	75.3 %
Age	68.7 (13.6)	70.3 (12.6)	72.2 (12.4)
Male	65.1 %	63.0 %	45.8 %
Hispanic	2.6 %	2.7 %	2.3 %
Non-Hispanic Black	3.5 %	2.7 %	2.8 %
Current Smoker	15.0 %	11.5 %	8.6 %
CKD (<60 ml/min/1.73 m^2^)	35.0 %	38.0 %	42.8 %
Any cardiovascular disease	93.1 %	90.8 %	84.1 %
Type 2 Diabetes	39.3 %	38.9 %	41.0 %
Hypertension	70.4 %	80.0 %	83.9 %
Systolic blood pressure (mmHg)*	121 (21)	125 (21)	130 (21)
Diastolic blood pressure (mmHg)*	72 (15)	71 (14)	70 (13)
Body Mass Index (kg/m^2^)Ɨ	29.1 (6.9)	30.2 (7.0)	31.6 (8.6)
Sacubitril/Valsartan	0.3 %	0.1 %	0.0 %
ACE/ARB	57.9 %	63.0 %	59.7 %
β-blockers	59.8 %	69.4 %	65.9 %
Diuretics	52.9 %	51.8 %	59.8 %
Aldosterone antagonist	11.8 %	9.5 %	6.3 %
Any HF-related medication	81.9 %	87.2 %	88.6 %
Statins	55.1 %	63.7 %	60.7 %
Glucose lowering drugs	28.2 %	28.6 %	28.9 %
*Percent missing: HFrEF 0.5 %, HFmrEF 0.7 %, HFpEF 0.3 %.			
ƗPercent missing: HFrEF 1.1 %, HFmrEF 0.9 %, HFpEF 0.8 %.			

For each of the eight outcomes, sample sizes, years of follow-up, and the number and percent with an event are displayed in Table 2. Mean follow-up time was marginally greater for HFpEF for each outcome except progression to insulin. The much larger size of the HFpEF group generated many more total events of all outcomes even when the percent with an outcome was lower. For example, 15.1 % of patients with HFrEF experienced a heart failure hospitalization, resulting in 368 events. In comparison, although 14.0 % of HFpEF patients experienced a heart failure hospitalization, this amounted to 1,742 individuals with such an outcome.

**Table 2:** Sample size, mean (SD) follow-up, and number and percent with an event for each of the eight major study outcomes by heart failure type.

	HFrEF			HFmrEF			HFpEF		
	Analysis		Mean (SD)	Analysis		Mean (SD)	Analysis		Mean (SD)
	Sample Size	Number (%)	Years of	Sample Size	Number (%)	Years of	Sample Size	Number (%)	Years of
Outcome	(% of Sample)	with Event	Follow-up	(% of Sample)	with Event	Follow-up	(% of Sample)	with Event	Follow-up
All-Cause Mortality (n = 16,516)	2,430	962	3.29	1,646	646	3.62	12,440	5,707	4.07
	(14.7 %)	(39.6 %)	(3.54)	(10.0 %)	(39.3 %)	(3.45)	(75.3 %)	(45.9 %)	(3.62)
Heart failure hospitalization (n = 16,516)	2,430	368	2.94	1,646	234	3.22	12,440	1,742	3.76
	(14.7 %)	(15.1 %)	(3.35)	(10.0 %)	(14.2 %)	(3.29)	(75.3 %)	(14.0 %)	(3.54)
Hospitalization for any cause (n = 16,516)	2,430	1,470	1.65	1,646	1,069	1.76	12,440	9,105	1.81
	(14.7 %)	(60.5 %)	(2.37)	(10.0 %)	(65.0 %)	(2.37)	(75.3 %)	(73.2 %)	(2.38)
Incident CKD^a^ (n = 7,731)	1,176	391	2.73	775	227	3.17	5,780	1,970	3.20
	(15.2 %)	(33.3 %)	(3.15)	(10.7 %)	(29.3 %)	(3.25)	(73.1 %)	(34.1 %)	(3.20)
Progression of eGFR Stage^b^ (n = 12,183)	1,678	811	2.07	1,169	569	2.29	9,336	4,898	2.37
	(13.8 %)	(48.3 %)	(2.71)	(9.6 %)	(48.7 %)	(2.72)	(76.6 %)	(52.5 %)	(2.65)
Progression of CKD^c^ (n = 4,452)	502	281	1.54	394	232	1.67	3,556	2,141	1.94
	(11.3 %)	(56.0 %)	(2.18)	(8.9 %)	(58.9 %)	(2.04)	(79.9 %)	(60.2 %)	(2.22)
Incident T2D^d^ (n = 9,520)	1,438	157	3.16	959	101	3.53	7,123	1,058	3.77
	(15.1 %)	(10.9 %)	(3.45)	(10.1 %)	(10.5 %)	(3.42)	(74.8 %)	(14.9 %)	(3.50)
Progression to insulin^e^ (n = 4,047)	627	133	2.30	427	103	2.68	2,993	742	2.07
	(15.5 %)	(21.2 %)	(2.86)	(10.6 %)	(24.1 %)	(2.98)	(74.0 %)	(24.8 %)	(2.50)

a Two eGFR values < 60 ml/min/1.73 m^2^ among those without CKD at baseline and available follow-up eGFR data.

b Progression from baseline eGFR category 1/2 to 3a or higher, or from 3a to 3b or higher, or from 3b to 4 or higher, or from 4 to 5.

c Progression from baseline eGFR category 3a to 3b or higher, or from 3b to 4 or higher, or from 4 to 5.

d Among those with no T2D at baseline and at least 12 months of available data prior to diabetes diagnosis.

e Among those with T2D at baseline and no prior insulin dispense.

### 3.1 Incidence rates and rate ratios

For all outcomes except incident T2D, patients with HFrEF had the highest incidence rate per 1,000 person-years, and HFpEF patients had the lowest incidence rates (Table 3). For example, HFrEF patients without CKD at baseline developed it at a rate of 151.0 per 1,000 person-years (95 % CI 137.7–166.9) vs 97.9 (93.4–102.7) among HFpEF patients. However, among those without T2D at baseline, HFpEF patients had higher T2D incidence than HFrEF patients (39.2, 36.8–41.6 vs 30.8, 26.2–36.2).

**Table 3:** Age and sex adjusted incidence rates (95% confidence intervals) for each of the eight major study outcomes by heart failure type.

	Age/sex Adjusted Incidence Rate per 1,000 person-years		
	(95 % Confidence Intervals)		
Outcome	HFrEF	HFmrEF	HFpEF
All-Cause Mortality	130.6	106.9	99.9
	(122.5–139.2)	(98.9–115.5)	(97.1–102.8)
Heart failure hospitalization	57.4	46.7	36.4
	(51.8–63.7)	(41.1–53.1)	(34.7–38.2)
Hospitalization for any cause	223.0	210.8	194.5
	(211.8–234.8)	(198.5–223.9)	(190.5–198.6)
Incident CKD	151.0	104.6	97.9
	(136.7–166.9)	(91.8–119.1)	(93.4–102.7)
Progression of eGFR Category	259.0	227.2	219.3
	(241.5–277.7)	(209.1–246.7)	(213.2–225.6)
Progression of CKD	359.1	345.8	311.6
	(319.0–404.1)	(303.9–393.6)	(298.7–325.1)
Incident T2D	30.8	27.5	39.2
	(26.2–36.2)	(22.6–33.5)	(36.8–41.6)
Progression to insulin	90.5	87.9	83.0
	(76.2–107.4)	(72.3–106.8)	(77.2–89.3)

**Fig. 1:**
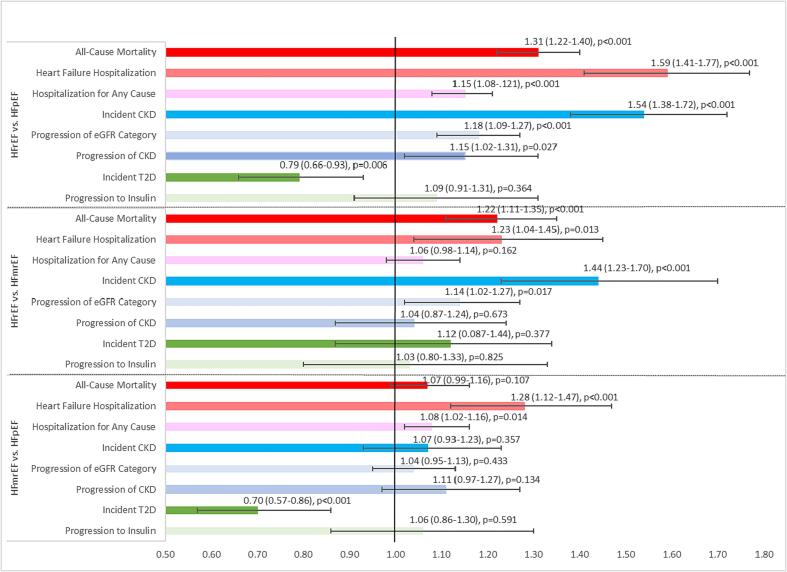
Age and sex adjusted rate ratios for incident rates of outcomes by EF categories.

Fig. 1 shows the rate ratios (95 % CI) comparing the adjusted incidence rates from Table 3 across each pair of HF types along with the p value for whether the rate is greater than 1. For all outcomes except T2D and progression to insulin, the rate ratios for HFrEF patients were significantly greater than for HFpEF patients, ranging from 1.15 (1.08–1.21, p < 0.001) for all-cause hospitalizations to 1.59 (1.41–1.77, p < 0.001) for heart failure hospitalizations. Incidence rates were 54 % higher for incident CKD (1.54, 1.38–1.72, p < 0.001), 18 % higher for progression of eGFR category (1.18, 1.09–1.27, p < 0.001), 15 % higher for CKD progression (1.15, 1.16–1.30, p = 0.027), and 31 % higher for all-cause mortality (1.31, 1.22–1.40) among HFrEF compared with HFpEF patients (p < 0.001 for all comparisons). However, T2D incidence was 21 % lower (0.79, 0.66–0.93, p = 0.006) in HFrEF versus HFpEF.

## 4 Discussion

In this longitudinal observational study of 16,516 HF patients with a HF diagnosis in their electronic medical record and an available echocardiogram, we found HFpEF accounted for over 75 % of the cases, but that on a per person basis, HFrEF generated significantly more morbidity and mortality. However, due to the much greater prevalence, the population health burden of HFpEF was about five to eight times larger. To our knowledge, these are findings that have not been previously reported. Our findings are also novel because we focus on patients with incident heart failure and include comorbidity outcomes that are not typically reported.

### 4.1 Comparison to previous studies

The high prevalence of HFpEF (75 % of patients with an EF) relative to HFrEF (15 %) is an important and somewhat surprising result as most studies conducted in patients hospitalized with HF reported a more balanced distribution of HFrEF and HFpEF. The ARIC study (Atherosclerosis Risk in Communities) examined heart failure hospitalizations from 2005 to 2014 in 4 US communities and found that 50 % had HFrEF compared with 39 % having HFpEF, but also reported that increasing trends in HF hospitalizations were primarily due to HFpEF.[7] In Olmstead County, 48 % of patients had HFpEF in 2000–2003, but that grew to 57 % in 2004–2007, again suggesting an increasing prevalence of HFpEF relative to HFrEF.[8] Older studies have consistently reported about 54 % of patients had HFrEF,[11], [12], [13], [14], [15], [16] although the Cardiovascular Health Study that focused on an elderly cohort (≥65 years of age) found 63 % of patients had HFpEF.[17] A previous study on the subset of patients with HHF in the EMR of KPNW found that 56 % of hospitalized HF patients had HFpEF vs 34 % with HFrEF.[18] We were unable to find contemporary published data on the distribution of HF types among general (non-hospitalized) HF patients, nor are such data reported in the current update of the American Heart Association’s Heart Disease and Stroke Statistics.[1] However, several community based studies using data through 2014 have consistently shown increasing trends in HFpEF coupled with decreasing trends in HFrEF.[7], [8], [9], [19] Such trends would therefore alter the distribution of HF types over time and could result in the imbalance we report herein. The ongoing Swedish Heart Failure Registry recently reported that about half of the population has HFrEF with one-quarter each having HFmrEF or HFpEF.[20] However, <10 % of *incident* cases are captured,[21] making comparison to our study of exclusively incident patients difficult.

In the current study, 32 % of patients initially selected for study did not have an echocardiogram in their EMR. Had these patients been tested, it is possible that the distribution of HF types would be different. However, even if all patients without an echocardiogram had HFrEF, which is unlikely, the proportion with HFrEF would still only be equal to those with HFpEF. As shown in the supplemental table 1, the baseline patient characteristics of the patients without an EF measure were more similar to those defined as HFpEF in terms of older age, more female, higher BMI and systolic blood pressure, and other factors, as compared with patients with HFrEF. Therefore, our data clearly suggest that HFpEF is the predominant type of HF.

Although reports are somewhat mixed, most studies have found equivocal outcomes in patients with preserved vs reduced ejection fraction.[19], [22], [23], [24] For example, a recent analysis from the Get With The Guidelines—Heart Failure found that among hospitalized patients, those with HFrEF had slightly higher risk for HF readmission, but lower all-cause readmission risk and equivalent 5-year mortality risk compared with HFpEF.[25] In contrast, with the exception of incident T2D, the rates of outcomes we report were consistently higher among those with HFrEF. In most cases, the differences were substantial, including a 59 % increase in HF hospitalizations, a 31 % increase in all-cause mortality, a 54 % in incident CKD and an 18 % and 15 % increase in eGFR category progression and progression of CKD, respectively. Furthermore, there was a clear association between ejection fraction and outcomes in that rates of outcomes for patients with HFmrEF invariably fell somewhere in between HFrEF and HFpEF. Thus, among this sample of general patients with HF, ejection fraction appears to be an important predictor of prognosis.

The annual global economic burden of heart failure was estimated at $108 billion in 2012, with costs in the U.S. accounting for nearly 30 % of that total.[26] Those costs are projected to increase by approximately 127 % by 2030.[27] Although there is no question that HF generates substantial resource utilization,[28] there does not appear to be any difference between HFrEF and HFpEF patients in per-person post-hospitalization resource use.[18] Nonetheless, due to the much higher prevalence of HFpEF, our current findings indicate the population-level burden of HFpEF is substantially greater than HFrEF, accounting for 5.9 times as many heart failure hospitalizations, 5.0 times as many incident cases of CKD, 7.6 times as many cases of CKD progression, and 6.7 times as many incident cases of T2D. Furthermore, the proportion of the total of each outcome exceeded the proportion of the sample accounted for by HFpEF. There is a strong need for HFpEF therapies to reduce the public health burden of heart failure.

### 4.2 Study limitations

Like most observational studies, ours has unavoidable limitations. Identifying HF cases from EMR data can result in mis-diagnosis, and several algorithms for doing so have been proposed. Our approach was appropriate for an epidemiological study in which complete ascertainment is important.[29] Nevertheless, our study population may include some individuals without validated HF. We adjusted our models for age and sex only because additional adjustment for available variables had little impact on the results. However, we cannot rule out residual confounding from variables that could not be assessed, although adjustment for several variables beyond age and sex had little impact on our findings. We used a single HF diagnosis and echocardiogram result to identify and classify patients with HF, which could result in misclassification or even inclusion of patients without clinical HF. The exclusion of patients without an echocardiogram likely eliminated many without validated HF. Furthermore, it has long been reported that HF is often present but not recognized.[30] It may be that the advent of EMRs and greater awareness of the HF epidemic has resulted in more aggressive diagnostic efforts. As previously mentioned, we excluded patients without an available echocardiogram which could have affected the relative differences between heart failure types. EF cut-offs for the categorization vary between guidelines, and we followed recommendations of the new universal definition of HF.[4] As we do not account for prior EF at inclusion to identify HFimprEF, the mid-range group does not differentiate between patients with HFpEF progressing to HFrEF and patients with previous HFrEF who have recovered (HFimprEF). Therefore, this group is not very well characterized, may at least partially explain the intermediate phenotype of this mrEF group in (some but not all) population-based studies, and hampers interpretation. Thus, we focused on the binary categorization of HFrEF and HFpEF in our discussion and conclusion. Measuring ejection fraction assessed from echocardiograms is imprecise, so some misclassification could have occurred. Finally, the study site is an integrated delivery system located in Portland, Oregon. Although the characteristics of its membership are representative of the service area,[31] the enrolled individuals are predominantly white with lower minority representation than in other parts of the country.

## 5 Conclusion

In conclusion, the presence of HF, regardless of HF types, is associated with poor clinical outcomes, lending further support to previous reports. We found that patients with HFrEF had poorer clinical outcomes at the person level than those with HFpEF. In contrast, HFpEF had a greater impact on the overall health care system due to a prevalence approximately-four times higher than that of HFrEF. Increasing trends in prevalence of HFpEF relative to HFrEF will only add to this public health burden,[7] underscoring the urgent need of effective HF therapies for patients with preserved ejection fraction.

## Declaration of Competing Interest

The authors declare that they have no known competing financial interests or personal relationships that could have appeared to influence the work reported in this paper: [Gregory A. Nichols reports financial support was provided by Boehringer Ingelheim GmbH, Qing Qiao reports employment by Boehringer Ingelheim GmbH, Anouk Deruaz-Luyet reports employment by Boehringer Ingelheim GmbH, Bettina J. Kraus reports employment by Boehringer Ingelheim GmbH].
