# Supplementary material for: The clinical burden of newly diagnosed Heart failure among patients with Reduced, mildly Reduced, and preserved ejection fraction
Source: Int J Cardiol Heart Vasc. 2023 Feb 14;47:101182. doi: 10.1016/j.ijcha.2023.101182 (PMC10424074; doi:10.1016/j.ijcha.2023.101182)
Supplement: Supplementary data 1 [file mmc1.docx]

Supplemental Figure 1. Flow diagram of sample selection.

|  |  |  |  |  |  |  |
| --- | --- | --- | --- | --- | --- | --- |
|  |  |  | HF Diagnosis 1/1/2005-12/31/19 n=37,773 |  |  |  |
|  |  |  | \|  \| \| --- \| |  |  |  |
|  |  |  | No Previous Mention of HF n=24,430 |  |  |  |
|  |  |  | \|  \| \| --- \| |  |  |  |
|  |  |  | Had Echocardiogram n=16,516 |  |  |  |
|  | \|  \| \| --- \| |  | \|  \| \| --- \| | \|  \| \| --- \| |  |  |
|  | HFrEF n=2,430 |  | HFmrEF n=1,646 |  | HFpEF n=12,440 |  |
|  |  |  |  |  |  |  |

Supplemental Table 1. Demographic and clinical characteristics of 24,430 patients with diagnosed heart failure by heart failure type.

|  |  | HF Type | | | |
| --- | --- | --- | --- | --- | --- |
|  |  | HFrEF: | HFmrEF: | HFpEF: | No EF |
|  | Total | EF < 40% | EF 41-49% | EF > 50% | Measurement |
| Baseline Assessment | (n=24,340) | (n=2,430) | (n=1,646) | (n=12,440) | (n=7,824) |
| % of Total | 100.0% | 10.0% | 6.8% | 51.1% | 32.1% |
| Age | 72.1 (13.4) | 68.7 (13.6) | 70.3 (12.6) | 72.2 (12.4) | 73.3 (14.7) |
| Male | 50.2% | 65.1% | 63.0% | 45.8% | 49.8% |
| Hispanic | 2.4% | 2.6% | 2.7% | 2.3% | 2.4% |
| Non-Hispanic Black | 2.7% | 3.5% | 2.7% | 2.8% | 2.2% |
| Current Smoker | 10.7% | 15.0% | 11.5% | 8.6% | 12.4% |
| CKD (<60ml/min/1.73m^2^) | 38.6% | 35.0% | 38.0% | 42.8% | 33.1% |
| Any cardiovascular disease | 90.6% | 93.1% | 90.8% | 84.1% | 100.0% |
| Type 2 Diabetes | 39.7% | 39.3% | 38.9% | 41.0% | 37.8% |
| Hypertension | 80.8% | 70.4% | 80.0% | 83.9% | 79.2% |
| Systolic blood pressure (mmHg)* | 129 (22) | 121 (21) | 125 (21) | 130 (21) | 131 (23) |
| Diastolic blood pressure (mmHg)* | 70 (14) | 72 (15) | 71 (14) | 70 (13) | 71 (14) |
| Body Mass Index (kg/m^2^)Ɨ | 31.0 (8.6) | 29.1 (6.9) | 30.2 (7.0) | 31.6 (8.6) | 30.6 (9.2) |
| Sacubitril/Valsartan | 0.0% | 0.3% | 0.1% | 0.0% | 0.0% |
| ACE/ARB | 65.9% | 85.4% | 77.0% | 64.3% | 60.0% |
| ß-blockers | 60.6% | 59.8% | 69.4% | 65.9% | 50.5% |
| Diuretics | 53.9% | 52.9% | 51.8% | 59.8% | 45.2% |
| Aldosterone antagonist | 6.0% | 11.8% | 9.5% | 6.3% | 3.1% |
| Any HF-related medication | 84.0% | 81.9% | 87.2% | 88.6% | 76.7% |
| Statins | 56.5% | 55.1% | 63.7% | 60.7% | 48.8% |
| Glucose lowering drugs | 27.6% | 28.2% | 28.6% | 28.9% | 25.3% |
| *Percent missing: HFrEF 0.5%, HFmrEF 0.7%, HFpEF 0.3%, No EF Measurement 6.5% | | | | |  |
| ƗPercent missing: HFrEF 1.1%, HFmrEF 0.9%, HFpEF 0.8%, No EF Measurement 5.8% | | | | |  |

Supplemental Table 2. Incidence rates (95% confidence intervals) for each of the eight major study outcomes by heart failure type, adjusted for age, sex, non-Hispanic Black race, smoking, history of atherosclerotic cardiovascular disease, blood pressure > 140/90, and use of HF medications.
